# A prospective study of the immune reconstitution inflammatory syndrome (IRIS) in HIV-infected children from high prevalence countries

**DOI:** 10.1371/journal.pone.0211155

**Published:** 2019-07-01

**Authors:** Mark F. Cotton, Helena Rabie, Elisa Nemes, Hilda Mujuru, Raziya Bobat, Boniface Njau, Avy Violari, Vidya Mave, Charles Mitchell, James Oleske, Bonnie Zimmer, George Varghese, Savita Pahwa, on behalf of the P1073 team

**Affiliations:** 1 Department of Pediatrics & Child Health, Stellenbosch University, Tygerberg, South Africa; 2 South African Tuberculosis Vaccine Initiative, Institute of Infectious Disease & Molecular Medicine and Division of Immunology, Pathology, University of Cape Town, Cape Town, South Africa; 3 Department of Pediatrics, University of Zimbabwe College of Health Sciences, Harare, Zimbabwe; 4 Department of Pediatrics & Child Health, University of KwaZulu-Natal, Durban, South Africa; 5 Kilimanjaro Christian Medical Centre, Moshi, Tanzania; 6 Department of Pediatrics & Child Health, Perinatal HIV Research Unit, University of the Witwatersrand, Johannesburg, South Africa; 7 BJ Medical College, Pune, India; 8 Department of Pediatric Immunology, University of Miami, Miami, FL, United States of America; 9 Department of Pediatrics & Child Health, Rutgers New Jersey Medical School, Newark, NJ, United States of America; 10 Frontier Science & Technology Research Foundation, Amherst, NY, United States of America; 11 Department of Pediatrics & Child Health, University of Miami Miller School of Medicine, Miami, FL, United States of America; Central University of Tamil Nadu, INDIA

## Abstract

**Background:**

The immune reconstitution inflammatory syndrome (IRIS) in HIV-infected infants and young children is relatively understudied in regions endemic for HIV and TB. We aimed to describe incidence, clinical features and risk factors of pediatric IRIS in Sub-Saharan Africa and India.

**Methods and findings:**

We conducted an observational multi-centred prospective clinical study from December 2010 to September 2013 in children <72 months of age recruited from public antiretroviral programs. The main diagnostic criterion for IRIS was a new or worsening inflammatory event after initiating antiretroviral therapy (ART). Among 198 participants, median age 1.15 (0.48; 2.21) years, 38 children (18.8%) developed 45 episodes of IRIS. Five participants (13.2%) had two IRIS events and one (2.6%) had 3 events. Main causes of IRIS were BCG (n = 21; 46.7%), tuberculosis (n = 10; 22.2%) and dermatological, (n = 8, 17.8%). Four TB IRIS cases had severe morbidity including 1 fatality. Cytomegalovirus colitis and cryptococcal meningitis IRIS were also severe. BCG IRIS resolved without pharmacological intervention. On multivariate logistic regression, the most important baseline associations with IRIS were high HIV viral load (likelihood ratio [LR] 10.629; p = 0.0011), recruitment at 1 site (Stellenbosch University) (LR 4.01; p = 0.0452) and CD4 depletion (LR 3.4; p = 0.0654). Significantly more non-IRIS infectious and inflammatory events between days 4 and 17 of ART initiation were noted in cases versus controls (35% versus 15.2%: p = 0.0007).

**Conclusions:**

IRIS occurs commonly in HIV-infected children initiating ART and occasionally has severe morbidity. The incidence may be underestimated. Predictive, diagnostic and prognostic biomarkers are needed.

## Introduction

CD4+ T cell depletion from untreated HIV infection predisposes to severe opportunistic and intercurrent infections [1] [2]. The first evidence that CD4 T cell recovery from antiretroviral medicines was associated with morbidity came from French et al who described unexpected *Mycobacterium avium-intracellulare* disease in immunosuppressed adults after commencing zidovudine [3]. After combination antiretroviral (ART) was introduced, this phenomenon, labelled as ‘immune restoration disease’ was increasingly recognized and ascribed to recovering pathogen-specific immunity [4]. Shelburne introduced the term “Immune Reconstitution Inflammatory Syndrome” (IRIS) recognizing that increased inflammation was a prominent feature [5]. Two IRIS presentations were recognized: a) “Paradoxical’ for worsening of a known inflammatory condition and b) “unmasking’ for a previously unrecognized infection [6].

Although IRIS is reported in children from diverse settings in HIV-infected (HIV+) children, only one pediatric prospective study from Thailand, addressed all forms of IRIS [7]. Four studies from Sub-Saharan Africa had prospective data collection. One was cross-sectional of children initiating ART within the previous two to 24 weeks in Uganda [8]. Another was a retrospective sub-analysis of IRIS events in the Nevirapine Resistance (NEVEREST) ART strategy trial [9]. The third addressed BCG IRIS adenopathy from the Children with HIV antiretroviral (CHER) trial [10]. Lastly, children with TB disease were followed for paradoxical TB IRIS once commencing ART [11].

The present study aimed to describe the incidence and clinical features of IRIS in ART-naïve HIV+ infants and young children where TB is prevalent and neonatal BCG immunization is routine. The study was designed to capture baseline clinical data associated with IRIS, determine the incidence of IRIS, to extend the spectrum of IRIS events, to better document IRIS morbidity and mortality and to determine whether IRIS affected short term ART outcomes.

## Methods

### Design

This prospective, observational clinical study was conducted in 7 clinical research sites: 3 in South Africa (Stellenbosch University [SU] Cape Town, the Perinatal HIV Research Unit [PHRU] Soweto and University of KwaZulu-Natal [UKZN] Durban, one each in Zimbabwe (University of Zimbabwe [UZ], Harare), Tanzania (Kilimanjaro Christian Medical Centre [KCMC], Moshi) and India (Byramjee Jeejeebhoy Government Medical College [BJMC], Pune). The ethics committees of all 7 clinical research sites approved the study and a parent or legal guardian of each participant gave written informed consent. All sites were in the International Maternal, Pediatric, Adolescent AIDS Clinical Trial (IMPAACT) network. Participants were recruited from nearby public programs where ART was initiated according to World Health Organization (WHO) 2010 Guidelines [12] in children with WHO Stage 3 and 4 and below 2 years of age. CD4 criteria below 5 years of age included a CD4 percentage ≤25% or absolute count <750 cells/mm^3^ and if older, <350 cells/mm^3^. Antiretroviral dosages were according to WHO-approved weight bands [12]. In South Africa, all infants below a year of age were eligible for ART [13].

ART-naïve HIV+ children from 4 weeks to 72 months of age were eligible. If below 12 months of age, BCG immunization was a requirement. Criteria for HIV diagnosis were positive virological tests from two samples at separate time points, including one from an accredited laboratory. Assays included HIV DNA PCR and plasma HIV RNA above 5000 copies/mm^3^. For children above 18 months of age, one assay could be a rapid HIV antibody test. Malignancy was an exclusion criterion. Maternal and/or paternal written informed consent and approval by ethics committees from participating sites were required.

The entry study visit was within 7 days preceding ART initiation and at weeks 2, 4, 8, 24 and 48 on ART. After IRIS recognition, additional visits were at weeks 1, 4, 16 and 24 thereafter. Blood samples for lymphocyte subsets and plasma HIV RNA were collected at enrolment, weeks 2 and 48 post ART initiation, and for IRIS cases, at all study visits.

### Identification and characterization of IRIS

**Fig 1:**
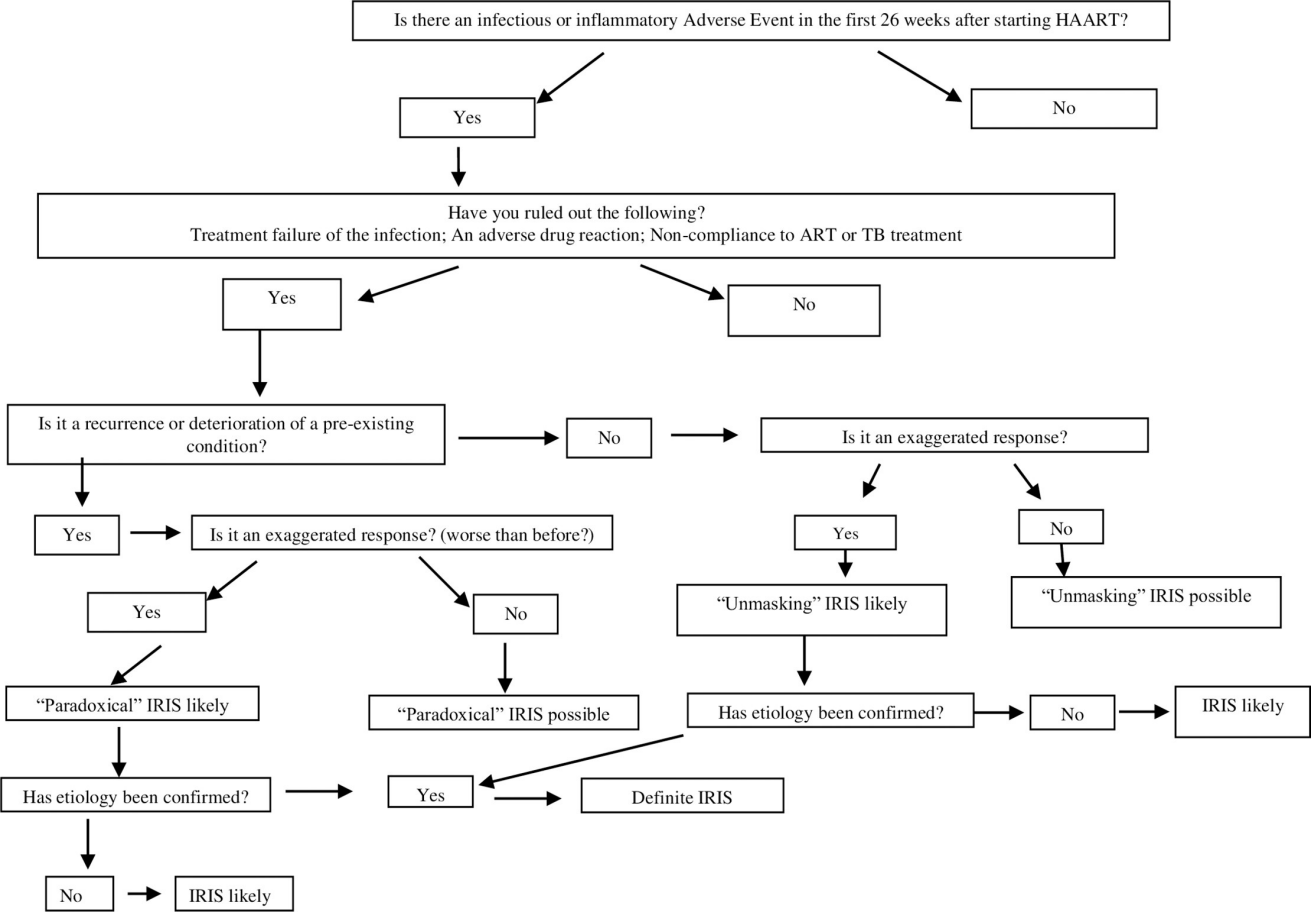
Algorithm for evaluating IRIS events.

At baseline, participants were examined with documentation of existing and pre-existing conditions. The BCG injection site and regional lymph node sizes were inspected so that changes suggestive of IRIS would be subsequently recognized. The diagnostic criteria for IRIS were based on those proposed by Haddow et al (Table 1 and Fig 1) [14]. The most important criterion was onset or worsening of an inflammatory event after ART initiation together with viral load reduction or CD4 recovery. All new or worsening inflammatory events were evaluated for IRIS. Site investigators received training on IRIS recognition and were encouraged to submit descriptions of suspected IRIS events to two investigators (MFC and HR). All clinical events were documented on case report forms and entered into the IMPAACT database. Also, clinical events were evaluated at approximately 3 monthly intervals in case IRIS events were not recognized. Site investigators were then requested to provide more information. An IRIS committee, comprising HIV specialists from the protocol team, evaluated all cases to determine if IRIS was present. The International Network for Study of HIV-associated IRIS (INSHI) diagnostic criteria were applied retrospectively for TB IRIS [15]. IRIS was designated paradoxical if an underlying condition was already known and unmasking if a new condition was identified. Dermatological IRIS was classified as paradoxical if already present at baseline. For children with baseline TB, an IRIS event ascribed to TB but at a different anatomical site, was considered ‘site unmasking’. BCG IRIS was considered paradoxical, due to residual *M*. *bovis*-BCG organisms or antigen at the injection site or ipsilateral axillary lymph nodes. Participants had study identification numbers (SID). IRIS events were designated as complicated, if associated with hospitalization or death.

**Table 1:** Criteria for diagnosing paradoxical and unmasking IRIS.

Unmasking IRIS
Clinical criteria 1. Temporal relationship: ART initiation precedes clinical deterioration 2. New onset of symptoms of an infectious or inflammatory condition after initiating ART 3. Consistent with the presence of pre-existing causative pathogen or antigen when starting ART 4. Either of the following: a. Onset within 3 months after initiating ART b. Atypical or exaggerated clinical, histological, or radiological findings in terms of severity, character of inflammatory response, rapidity of onset, or localization 5. Exclusion of other causes 6. Events not explained by: a. Expected clinical course of another condition b. Drug toxicity c. Newly acquired infection, based on clinical history or other evidence d. Failure of ART: presumptive, based on either nonadherence or resistance to ART, or confirmed, based on VL assay if available

### Statistical analysis

Comparisons of baseline characteristics were performed using univariate Wilcoxon and Fisher’s Exact tests. Multivariate nominal logistic regression for relationship of IRIS to baseline characteristics included factors with p-value ≤ 0.05. When considering similar variables, for example CD4 T-cell count or percentage, the variable with the lowest p-value was selected. The degree of association was reported by odds ratios (OR). New onset infectious or inflammatory events, not considered IRIS, were documented from day 4 on ART. As all participants were seen at week 2 and allowing a window of 3 days, the frequency of these events between days 4 and 17 was compared in those with and without IRIS. For end of study evaluations, children completing a minimum of 20 weeks were included. Analysis was done using JMP 14.1, SAS Corporation. To investigate the relationship between IRIS and other inflammatory or infectious events, an on-line calculator for Chi-Squares and Fisher’s Exact tabulation was used (http://www.graphpad.com). We used WHO criteria to exclude outliers for anthropometry Z-scores. For height for age we excluded Z-score <-5 and >+3; for weight for age we excluded Z-scores <-5 and >+5 and for weight for height Z-scores we excluded Z-scores < -4 and >+5 [16].

## Results

### Baseline

The study was conducted between December 2010 and September 2013. Nine of 207 participants were excluded for the following reasons: two parents withdrew consent, two children received no ART, one was ART-ineligible by concurrent guidelines and 4 participants only attended the baseline visit. Baseline demographics of the 198 participants evaluated for IRIS are in Table 2. The majority came from SU (39.6%) and UZ (25.2%). Median age was 1.15 years (0.48–2.21) with 91 participants (45%) below one year of age. Stunting was common with relatively well-preserved weight for height Z-scores. The majority (61%) had WHO stage 3 or 4 disease. TB was common, 21% having previous TB, 17% receiving anti-TB treatment and 6% isoniazid prevention therapy (IPT). Twenty-six participants (13%) were receiving corticosteroids either topically or systemically. CD4 counts and percentages were relatively well preserved. The majority (85%) had ≥5 log HIV RNA copies/mm^3^. The ART regimens are outlined in S1 Table. The most common regimen was lopinavir-ritonavir plus abacavir and lamivudine (57.6%) followed by nevirapine plus abacavir and lamivudine (25.3%).

**Table 2:** Baseline characteristics associated with IRIS.

	All	No IRIS	IRIS	Univariate	Multivariate logistic regression*					
Number	198	160	38							
					All IRIS		Paradoxical IRIS (n = 27)		Unmasking IRIS (n = 11)#	
				P-value	OR (95% CI)	P-value	OR	P-value	OR	P-value
**Site**										
SU versus other sites				0.0004	0.395 (0.158–0.987)	0.0468	0.77 (0.259–2.291)	0.639	0.106 (0.0187–0.604)	0.0115
1. SU	79 (39.9%)	54 (33.8%)	25 (65.8%)							
2. PHRU	19 (9.5%)	19 (11.9%)	0 (0%)							
3. UKZN	25 (12.6%)	22 (13.8%)	3 (7.9%)							
4. UZ	50 (25.2%)	42 (26.3%)	8 (21.1%)							
5. KCMC	20 (2.5%)	19 (11.9%)	1 (2.6%)							
6. BJMC	5 (2.5%)	4 (2.5%)	1 (2.6%)							
Median (IQR) age (years)	1.2 (0.5;2.3)	1.3 (0.6;2.3)	0.7 (0.3;1.8	0.0239						
<1 year of age	90 (45.5%)	66 (41.3)	24 (63.2)	0.018	1.642 (0.584–4.619)	0.347	3.868 (1.088–13.75)	0.037	0.25 (0.045–1.37)	0.063
Male	104 (52.5%)	82 (51.3%)	22 (57.9%)	0.477						
Median (IQR) WAZ	-1.65 (-2.89;-0.65) n = 178	-1.58 (-2.81;-0.50) n = 144	-1.91 (-3.19;-1.15) n = 34	0.176						
Median (IQR) HAZ	-1.54 (-2.28;-0.64) n = 188	-1.51 (-2.26;-0.60) n = 154)	-1.80 (-2.47;-0.84) n = 34	0.284						
Median (IQR) WHZ	-0.80 (-2.09;0.08) n = 178	-0.80 (-1.96;0.81) n = 143	-0.71 (-2.53;0.08) n = 35	0.85						
WHO Stages										
Stage 1	45 (23%)	41 (25.9%)	4 (10.5%)	0.136						
Stage 2∏	30 (15.3%)	25 (15.8%)	5 (13.2%)							
Stage 3	100 (51%)	77 (48.7%)	23 (60.5%)							
Stage 4	21 (10.7%)	15 (9.5%)	6 (15.8%)							
Stage 1 versus 2,3,4				0.053						
TB prior to baseline	42 (21.2%)	28 (17.5%)	14 (36.8%)	0.0141						
IPT at baseline	12 (6.1%)	10 (6.3%)	2 (5.26%)	1						
Current anti-TB treatment	33 (16.67%)	20 (12.5%)	13 (34.2%)	0.0029*	0.336 (0.11–1.024)	0.0551	0.259 (0.067–0.999)	0.0498	0.851 (0.185–3.916)	0. 836
Corticosteroids										
1. Any (topical or systemic) corticosteroids	26 (13.1%)	15 (9.4%)	11 (29%)	0.0025	0.452 (0.56–1.315)	0.145	0.506 0.153–1.672)	0.264	0.489 (0.103–2.32)	0.368
2. Systemic only	10 (50.1%)	4 (1.9%)	6 (15.8%)	0.004						
CD4+% (Median/ IQR)	19.6 (13.9;27.3) n = 194	20.1 (14.6;27.7) n = 157	16.7 (11.8; 21.7) n = 37	0.0152						
CD4+ (cells/mm^3^) (Median/IQR)	1068(599; 1784)	1188 (618; 1862)	838 (223; 1259)	0.0115	10.609 (0.758–148.55)	0.08∉	57.62 (1.997–1662)	0.0181∉	0.195∉(0.004–12.299)	0.411
Plasma HIV-RNA (Log_10_ copies/mm^3^) (Median / IQR) Ø	5.91 (5.33; 6.47)	5.8(5.24; 6.34) Ø	6.32 (5.84; 6.81)	<0.0001	0.0093 (0.0005–0.189)	0.0023∉	0.0033 (0.00008–0.1426)	0.0029∉	0.212 (0.003–12.966∉	0.47

OR–Odds ratio (*For IRIS = Yes, odds of No versus Yes); P-values for full model on multinomial logistic regression: All IRIS–p <0.0001; paradoxical IRIS–p <0.0001; unmasking IRIS–p = 0.053

∉ P-values derived from parameter estimates

CI–Confidence interval; IQR–interquartile range; WAZ–weight for age Z-score; HAZ–Height for age Z-score; WHZ–weight for height Z-score; IPT–isoniazid prevention treatment

# SID 1228 site unmasking TB IRIS

∏ WHO staging not assigned in 2 without IRIS.

Ø 8 plasma HIV RNA levels from baseline unavailable in participants without IRIS.

### IRIS

Thirty-eight participants (18.8%) developed 45 IRIS episodes. Median time to first IRIS event was 21 days (IQR 13.5 to 55) (range: 4 to 105 days). Sixteen episodes (35.6%) occurred in the first 14 days of ART and 7 (15.6%) after day 60. Five participants (12%) with baseline CD4 ≥ 25% and 4 (10%) in WHO Stage 1 developed IRIS.

**Fig 2:**
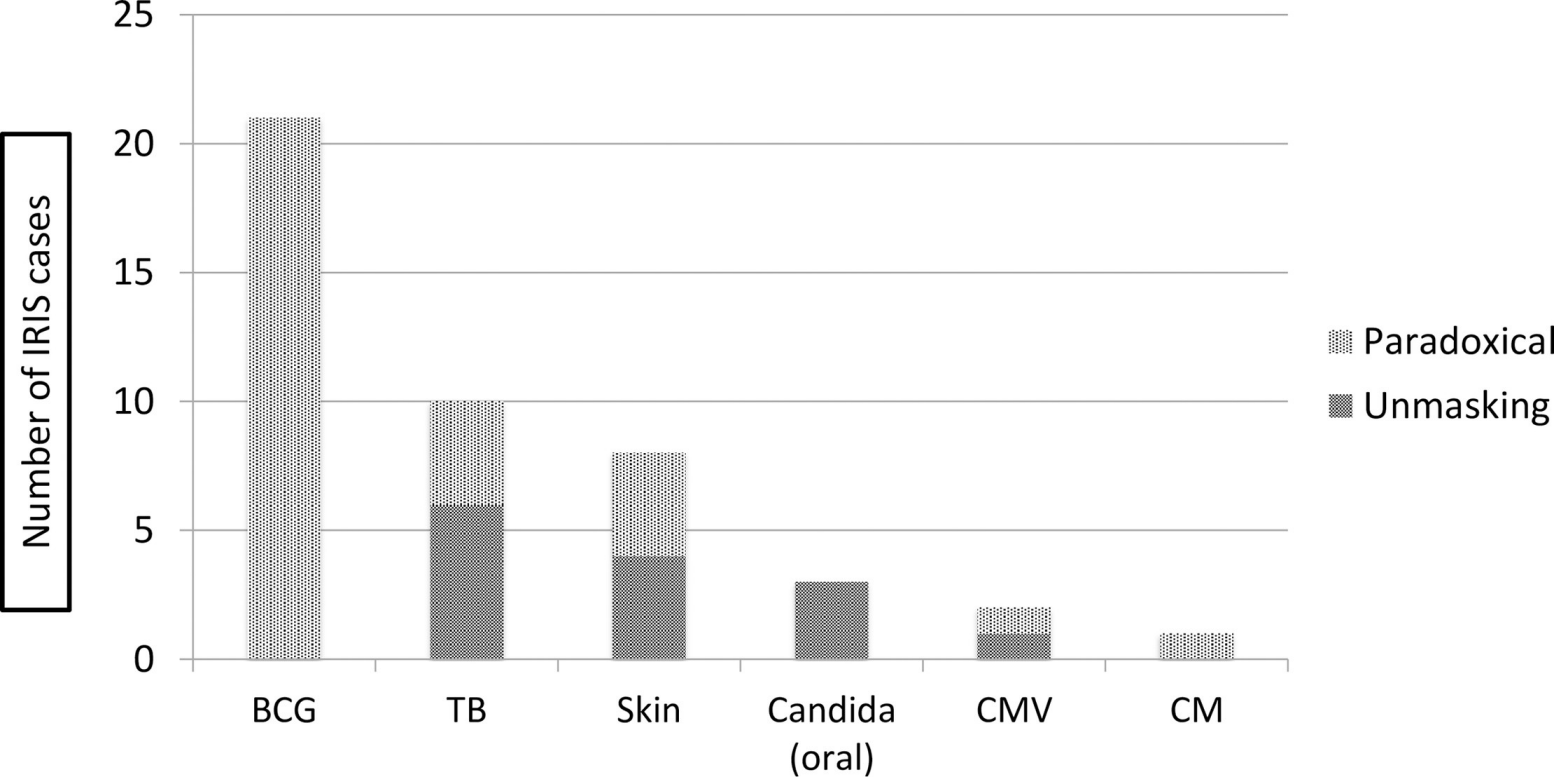
Types of paradoxical and unmasking IRIS. In dermatological IRIS, 1 case of Zoster IRIS was considered unmasking. BCG–Bacille Calmette Guérin, TB–tuberculosis, CMV–cytomegalovirus, CM–cryptococcal meningitis.

**Fig 3:**
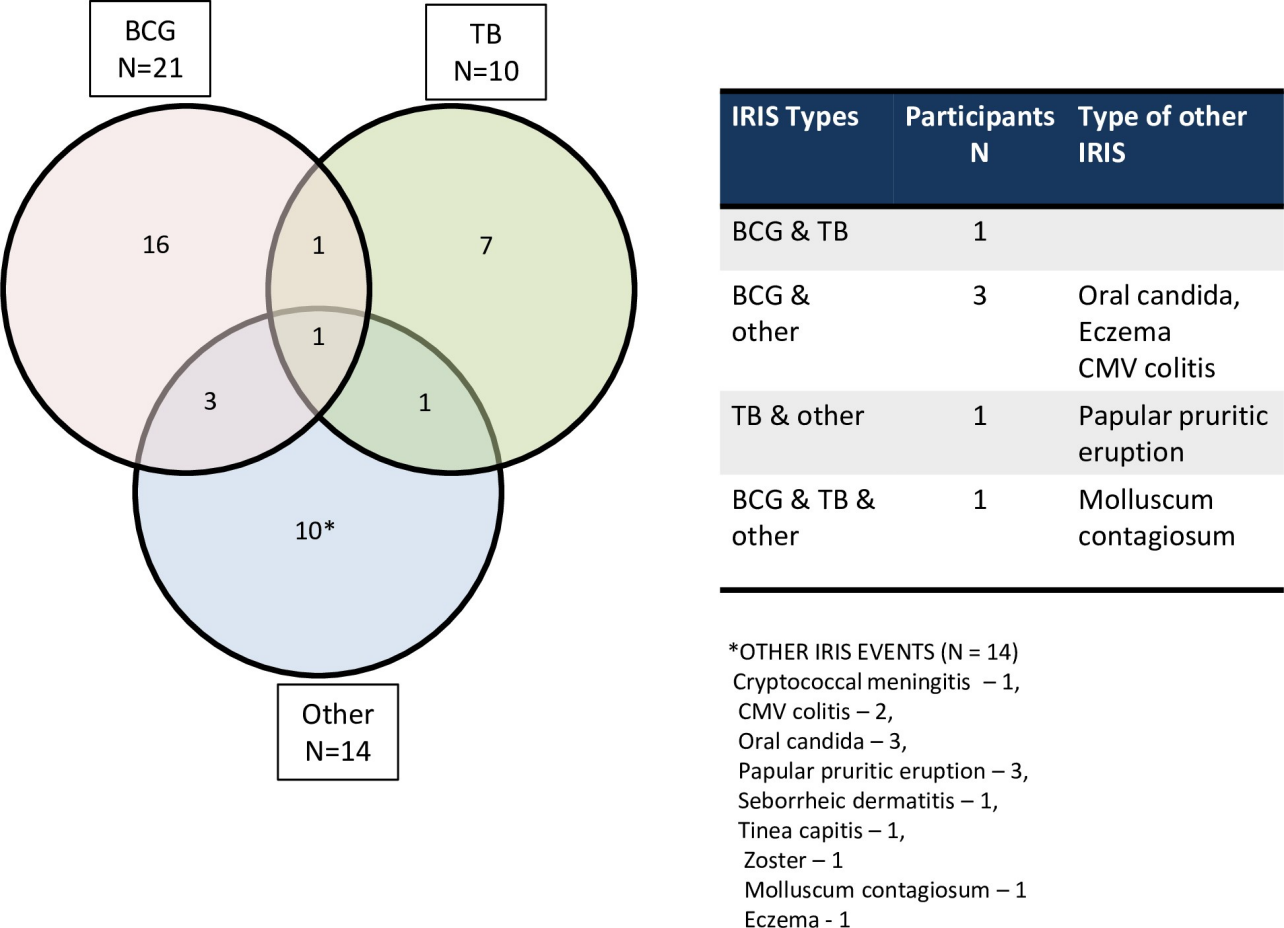
Distribution of IRIS events in participants.

Paradoxical IRIS comprised 71% of IRIS episodes (Fig 2). Of these, most were BCG-related, (21/46.7% episodes), followed by TB (10/22.2%) and infective or inflammatory skin conditions (9/26.5%) (Fig 2). Of 6 participants (15.8%) with multiple IRIS events, 5 had 2 events and one had 3 events (Fig 3). Of these, two had both paradoxical and unmasking events and 4 children had all either unmasking or paradoxical. Five dermatological or oral IRIS events were diagnosed retrospectively after reviewing the clinical data (oral candidiasis, tinea capitis, papular pruritic eruption, zoster and seborrheic dermatitis) For 42 events from 32 participants, reviewed retrospectively, we could not diagnose IRIS as were unable to determine whether there was increased or ‘excessive’ inflammation.

Apart from a child aged 16 months, BCG IRIS occurred in infants below a year of age. Its spectrum included local injection site reactions, regional axillary adenopathy and both in combination (S2 Table and S1 Fig). Most TB IRIS (7/10) was unmasking and identified at SU (8/10). (See TB IRIS diagnosis by INSHI criteria in S3 Table) One participant (SID 1228), treated for pulmonary TB, developed seizures on day 13 due to an unmasked intracerebral tuberculoma (S2 Fig).

### Factors associated with IRIS

On univariate analysis, being recruited at SU, younger age, not WHO stage 1, a history of TB, receiving anti-TB treatment, receiving topical or systemic corticosteroids, a higher HIV viral load and CD4 depletion were associated with IRIS (Table 2). The ART regimen was not associated with IRIS (S1 Table)

By multinomial logistic regression, IRIS was most associated with elevated plasma HIV RNA (LR 10.629; p = 0.0011) and recruitment at SU (LR 4.01p = 0.0452). Low CD4 T-cell count (LR 3.4; p = 0.065) and receiving anti-TB therapy (LR 3.674; p = 0.055) approached significance. For paradoxical IRIS, plasma HIV RNA was even more strongly associated (LR 12.556; p = 0.0004). Current TB treatment (LR6.244; p = 0.00125), age below a year (LR 5.559; p = 0.0184) and a lower CD4+ T-cell count (LR 5.35; p = 0.0207) were also significantly related. For unmasking IRIS, only being at SU was significantly associated (LR 7.993; p = 0.0047) (Table 2).

### Events during study and impact of IRIS on outcomes

Two children were lost to follow-up after two weeks. The median duration for BCG IRIS was 22 weeks (S2 Table). Fifteen participants commenced anti-TB treatment on study, including 6 with unmasking TB IRIS. There was a trend for those with IRIS unrelated to TB to start anti-TB treatment (p = 0.17) (Table 3). IRIS was implicated in one of three deaths of children with IRIS (S4 Table). Including the one fatality, 7 participants (18.4%) had severe IRIS (S5 Table). These included 4 of 10 with TB IRIS. SID 1302 died of progressive paradoxical IRIS. Three participants had unmasking TB IRIS: SID 1228 (S2 Table), on anti-TB treatment at baseline had seizures due to unmasked intracerebral tuberculomas (S2 Fig); SID 1246 developed gall bladder destruction, requiring a porto-jejunostomy. For SID 1652, the diagnosis of TB meningitis was supported by magnetic resonance imaging showing intracerebral granulomas and eventual resolution on anti-TB meningitis therapy (S3 Fig). Two participants (SID 1255 and 1282) developed severe CMV colitis IRIS, one unmasking and one paradoxical, requiring intensive care support. SID 1303 presenting with inability to walk, required hospitalization for cryptococcal meningitis IRIS. Cerebrospinal fluid cryptococcal antigen was detected at initial presentation but not at IRIS diagnosis.

**Table 3:** Events during study.

Characteristic#	All (N = 198)	No IRIS (N = 160)	IRIS present (N = 38)	P value
During study				
Weeks on study (IQR)	48 (28.5; 48)	48 (24; 48)	48 (45–49)	0.165
Commenced IPT	3 (1.5%)	2 (1.25%)	1 (2.63%)	0.7
TB				
Began TB treatment	15 (7.4%)	6 (3.75%)	9 (23.7%)§	0.0003*
TB IRIS			6 (15.8%)	
Other IRIS (including BCG)			2 (5.3%)	0.17
Death	9 (4.5%)	6 (3.8%)	3 (7.9%)	0.378
Non-IRIS infectious or inflammatory events from day 4 to day 17	40 (20.2%)	24 (15.2%)	14 (35%)	0.0007*

# 4 children lost to follow-up after baseline visit excluded

* Fisher’s exact two-tail test

§ 6 of 9 in IRIS group commencing anti-TB therapy had unmasking TB IRIS; 3 participants had non-TB IRIS events–oral candidiasis; BCG and oral candidiasis; and CMV colitis

Events at the end of the study in those completing at least 20 weeks on study are shown in Table 4. During the study, 205 infectious or inflammatory events not considered IRIS were documented in 101 (51%) children (S4A Fig), mainly dermatological, respiratory and gastrointestinal (S4B Fig), 70% noted in the first 60 days. Significantly more events were noted in children from SU (65.8%: 52/79) than from other sites (41.2%: 49/119) (p = 0.0006). As IRIS participants had more visits and more opportunities for documenting intercurrent events, the comparison was restricted to the first 17 days on ART, when frequency of study visits was the same. Forty children developed 49 non-IRIS infectious or inflammatory events, significantly more common in those with IRIS (p = 0.0007) (Table 4) but similar for all sites (SU: (19 / 9.6% versus 21 / 10.6% from other study sites; p = 0.284).

**Table 4:** Anthropometry, CD4 data and HIV viral loads at study end for those completing ≥20 weeks on study.

	N = 170	N = 136	N = 34	
WAZ median (IQR)	-1.07 (-1.92;-0.09) n = 168	-1.06 (-1.78; -0.07) n = 135	-1.25 (-2.44;-0.44) n = 33	0.28
HAZ median (IQR)	1.31 (-1.94; -0.49) N = 168	-1.31 (-2.11; -0.48) n = 134	-1.35 (-1.81; -0.50)	099
WHZ median (IQR)	-0.16 (-1.05; -0.52) n = 166	-0.17 (-1.03;0.50) n = 133	-0.11 (-1.34;0.66) n = 33	0.67
CD4+ %	30.3 (24.1; 36.5)	31 (25.3; 37.9) n = 119	27.4 (20.75; 32.02) n = 34	0.0173
CD4+ cells/mm^3^	1598 (1195; 2201)	1582 (1168; 2194)	1679 (1366; 2216)	0.49
Plasma HIV-RNA Log_10_copies/mm^3^	1.96 (1.7; 3.1)	1.94 (1.7; 3.09)	2.3 (1.7: 3.67)	0.47
Plasma HIV-RNA <400 copies/mm^3^	91(53.5%)	101 (64.3%)	28 (68.3%)	0.39
**Change between baseline and study end in those ≥20 weeks on study**				
WAZ	0.60 (-0.27;1.65) n = 169	0.42(-0.3;1.76) n = 124	0.74(0.17;1.52)	0.27
HAZ	0.25 (-0.17;0.83) n = 159	0.24(-0.22;0.7) n = 128	0.48(0.03;1.31) n = 31	0.087
WHZ	0.42 (-0.57;2.07) n = 153	0.49(-0.56;2.06) n = 122	0.35(-1.07;2.11) n = 31	0.69
CD4+ %	8.5 n = 150	8 (3.13;14) n = 116	9.25 (4.7; 13.63) n = 34	0.90
CD4+ cells/mm^3^	498 (16.3; 1071)	372 (-17,5; 971)	868 (320; 1352)	0.015
Plasma HIV-RNA Log_10_copies/mm^3^	3.59 (4.22; 2.4)	3.49 (-4.14; -2.38)	4.01 (-4.51; -2.75)	0.052

CD4, viral load and anthropometric measures were assessed in 170 children followed for 20 weeks (Table 4). Anthropometry was similar between those with and without IRIS. The CD4 percentage in those with IRIS was still significantly below those without IRIS but absolute CD4+ T cell counts were similar. The CD4+ T cell count increase was significantly higher in those with IRIS, suggesting greater recovery. There was no difference in virological suppression although the IRIS group experienced a greater decline in viral load. Participants with IRIS had a higher increase in height for age Z-score.

### IRIS management

Six participants were already receiving prednisone when IRIS was diagnosed. Prednisone was used to manage severe IRIS in 6 participants, one of whom (SID 1302) died (Table 4). BCG IRIS was managed conservatively, although two children required pus aspiration for symptomatic relief (S2 Table).

## Discussion

This is the first multi-centre study of IRIS in infants and young children from Sub-Saharan Africa and India that prospectively evaluated for both unmasking and paradoxical IRIS. We confirmed many findings noted individually in other paediatric studies and provided some new insights [7–9, 17, 18].

### Frequency, spectrum and time of onset

Frequency and spectrum of IRIS events were similar to other large pediatric cohort studies where clinical data was collected prospectively [7–9]. Median time of first IRIS recognition was 21 days in our study, similar to the NEVEREST and Thai studies [8, 10] and in adults [19]. Earliest time for IRIS in our study was day 4, versus day 7 in NEVEREST and day 14 in Thailand [7, 9]. Over 15% of children in our study had more than one IRIS event versus 6.9% of Thai children [7] and 23.5% from NEVEREST [9]. Time to IRIS onset could not be assessed in Ugandan pediatric study due to its cross-sectional design [19].

BCG IRIS was most common, followed by TB, dermatological, oral candida, CMV and cryptococcal meningitis. The highest frequency and co-occurrence of BCG and TB IRIS was also documented in the NEVEREST study [9]. We found paradoxical IRIS more commonly than unmasking, most likely due to BCG IRIS being the most common and easily recognized IRIS. In the cross-sectional Ugandan study, the only other pediatric cohort study to differentiate paradoxical from unmasking IRIS, unmasking IRIS mainly from bacterial pneumonia and dermatological infections, was three times higher than paradoxical IRIS. This study is limited by not prospectively documenting existing conditions at baseline. For TB IRIS, both presentations were equally represented. BCG IRIS comprised only 4.8% of IRIS cases in infants between 6 and 12 months of age, 10% of this study population. [9] In a large prospective IRIS study in adults with advanced HIV disease in South Africa, unmasking IRIS, mainly due to unrecognized TB, was twice as common as paradoxical IRIS [19]. In our study, unmasking TB IRIS (6 cases) was slightly more common than paradoxical, probably reflecting both active screening for TB at all of the sites but also the difficulty in diagnosing TB in HIV+ children.

In our study, inspection of the BCG injection site and assessing the right axillary lymph nodes for increased size and tenderness was standard. We provided accurate data on BCG IRIS, showing that local BCG IRIS was the most common variant (9 of 21: 43%). In comparison, in the NEVEREST cohort, local BCG IRIS comprised 12% of BCG IRIS, most likely as although data were recorded prospectively, IRIS assignation was retrospective [9]. We described for the first time the median duration of BCG IRIS (22: 10.9–31 weeks), important for clinicians when counselling parents. BCG IRIS rarely occurs in older children, often associated with BCG re-immunization [20]. In the CHER trial we reported BCG IRIS adenitis as less common in infants with high CD4 percentages commencing early ART rather than deferred until CD4 depletion triggered ART initiation. In a subset of infants with CD4 < 25% beginning early ART, the frequency of BCG IRIS adenitis was similar to the deferred arm [21]. In the present study, the median age of ART initiation was 21.6 weeks, similar to the deferred arm of the CHER trial.

For TB-related IRIS, TB was confirmed by culturing drug sensitive *Mycobacterium tuberculosis* in two cases (20%). In the remainder, diagnosis was based on contact history, suggestive chest radiographs or brain magnetic resonance imaging, (MRI) and positive Mantoux skin tests. Unmasking of a brain tuberculoma through new onset seizures, was previously noted in a Cape Town case series from SU [22]. SID 1652, although diagnosed with TB meningitis IRIS, had little supporting evidence (S5 Table). Cerebrospinal fluid showed a neutrophil predominance, atypical for TB meningitis with normal protein and glucose levels. The MRI showed multiple intracerebral granulomas with ring-enhancing oedema. This child improved on anti-TB meningitis therapy. One case of unmasking TB IRIS was diagnosed through a strongly positive Mantoux skin test and the mother’s chest radiograph being compatible with pulmonary TB. This infant developed progressive biliary obstruction, possibly due to expanding lymph nodes in the porta hepatis requiring surgical repair and hospitalization for 6 months. Also, we observed paradoxical TB IRIS in 3 of 33 (9%) with active TB at baseline, more common than the 1.9% (2 of 104) from a prospective study of children already on anti-TB therapy at ART initiation [11].

In our series, dermatological IRIS comprised 24.5% of events, similar to the older Ugandan children [8]. Manifestations included papular pruritic eruption, seborrheic dermatitis, zoster and fungal infections. In the Thai study, the most commonly documented cutaneous IRIS events were viral, mainly zoster and herpes simplex [7]. In the NEVEREST study, only one case of seborrheic dermatitis IRIS and one of herpes labialis IRIS were noted (5.9% of IRIS events). As IRIS was not determined prospectively, dermatological events may have been missed [9].

We identified two children with CMV-related colitis IRIS, one unmasking and one paradoxical. CMV colitis IRIS is described in adults [23]. CMV-related IRIS featured prominently in early adult studies from Australia and the United States [4, 5] but not in Sub-Saharan Africa [19, 24]. One case of CMV IRIS pneumonitis was identified in the NEVEREST cohort [9]. The only other descriptions of CMV IRIS in children include two cases of retinitis [25] and one of fatal myoclonus-opsoclonus and cardiomyopathy from SU, Cape Town [26].

Cryptococcal meningitis, although uncommon, occurs in HIV+ children. [27] Paediatric cryptococcal IRIS was reported in the Thai study [7] and also in Cape Town [28].

### Baseline factors associated with IRIS

IRIS, although more common with advanced HIV disease, was noted in milder HIV infection [9, 18]. We found, on multinomial analysis, that baseline elevated HIV RNA viral load was significantly associated with IRIS. For paradoxical IRIS both baseline elevated HIV RNA and CD4 depletion were significantly related as already noted for BCG IRIS adenitis in the CHER trial [21]. In the 3 main pediatric IRIS cohort studies, CD4 cell depletion rather than viral load was associated with IRIS on multivariable analysis. In NEVEREST, CD4 depletion and low WAZ were risk factors on multivariable analysis [9]. Our prospective data confirm the importance of elevated viral load more so than CD4 depletion being associated with IRIS. In our study, low WAZ played no role.

Both high viral load and severe CD4 T-cell depletion were associated with paradoxical IRIS in a South African adult study [19]. In our study, being on current anti-TB therapy was significantly associated with paradoxical IRIS, most likely due to paradoxical TB IRIS. Another explanation is that isoniazid and rifampicin have activity against *Mycobacterium bovis*-BCG and might promote antigen release. Van Rie documented a low incidence (5 of 104; 4.8%) of paradoxical TB IRIS in children with a similar baseline profile to our study. She observed that some children fulfilling criteria for paradoxical TB IRIS probably had alternative diagnoses such as bacterial pneumonia as they responded to antibiotic therapy [11].

The topical corticosteroids reflect a high prevalence of dermatitis. Of note, although corticosteroids can prevent or ameliorate IRIS in adults, it did not prevent IRIS in our study. In a recently reported adult study, corticosteroids reduced the incidence of TB IRIS [29].

The only baseline factor associated with unmasking IRIS in our study was enrolment at SU. We ascribe this to more familiarity with IRIS at SU and proximity to the in-patient setting, enabling closer monitoring of participants. All children received a baseline chest. From the adult study from KwaZulu-Natal, associations included C-reactive protein above 25 mg/L, lower haemoglobin, more weight loss and radiological evidence of lymphadenopathy. The 2 most common causes of unmasking IRIS were TB and folliculitis, representing 11% and 19.3% of all IRIS cases respectively [19]. Both conditions are associated with elevated C-reactive protein [30] [31].

### Events during study and impact on IRIS outcomes

Severe morbidity was associated with suspected or confirmed TB IRIS and also CMV, affecting 18% of children with IRIS. Both unmasking and paradoxical TB IRIS had significant morbidity, with the only death related to paradoxical TB IRIS. Except for one retrospective study which documented severe IRIS morbidity and mortality [32] and isolated case reports, [10, 26] there has been little data on IRIS severity in children.

We observed a high frequency of intercurrent non-IRIS infectious and inflammatory events declining over time on ART. Many of these events were considered for IRIS but lacked information on the extent of disease and whether exacerbated by ART. Documentation of intercurrent events occurred significantly more commonly at SU. However, in the first 17 days on ART, when all participants had the same number of visits, there was no difference between the study sites. This finding suggests firstly, that despite our efforts, IRIS events may have been missed and that multiple IRIS events occur more commonly in those predisposed to IRIS. That TB treatment was more commonly initiated in those with IRIS is likely due to unmasking TB IRIS in 6 children. A similar high frequency of intercurrent infections was also observed in the prospective adult study from KwaZulu-Natal, but was not stratified by IRIS [19].

Although by the end of the study, the CD4 percentage in those with IRIS was still lower than those without IRIS, the latter had a greater increase in CD4 T-cell numbers. Those with IRIS had a greater fall in plasma HIV VL but virological suppression to below detectable limits were the same in those with and without IRIS. Poorer virological response of those with IRIS in the NEVEREST study was most likely related to rifampicin-induced lowering of lopinavir-ritonavir exposure [33].

### Management

BCG IRIS was not treated in our study. In earlier studies, anti-mycobacterial therapy was used often [9, 20]. reducing lopinavir-ritonavir and nevirapine exposure to rifampicin interactions [34, 35]. Screening for TB in endemic settings before ART initiation should reduce unmasking TB IRIS, as illustrated in Uganda where a TB screening program was associated with a 70% decline in incident TB [36]. Although there was high awareness for TB, screening practices were not uniform in our study and are not very sensitive in HIV-infected children. Many children have abnormal chest radiographs in the absence of TB [37]. Tuberculin skin tests are insensitive and microbiological culture has a low yield in children [38]. This was illustrated by finding 5 cases of unmasking TB IRIS at SU despite baseline screening.

### Limitations of the study

More IRIS cases and more intercurrent infections were identified at one site, most likely due to increased awareness, the practice of obtaining chest radiographs on all children commencing ART and close proximity to hospital wards, the latter facilitating regular monitoring of hospitalized children. That those with IRIS had more infectious or inflammatory events not regarded as IRIS, suggests that IRIS events were missed. Also, from cases reviewed retrospectively for IRIS, only 5 of 47 infectious or inflammatory events were considered IRIS cases, because of insufficient supporting data. Our inclusion of these retrospectively determined IRIS events is another limiting factor. Apart from BCG IRIS, which is easily recognized, IRIS diagnosis requires clinical skill. Although training took place, it may have been insufficient. A consideration for future studies is digital image capturing at baseline and thereafter to better diagnose dermatological and mucosal IRIS events and baseline exclusion of TB, including chest radiographs. Recognition of CMV IRIS required the availability of CMV viral load assays, which were not routinely performed even when available at sites.

### Relevance of the study

Although the study was completed 5 years ago and HIV guidelines have changed, the potential for IRIS still remains. Early infant diagnosis guidelines have already shifted from 6 weeks of age to birth, with the potential to begin ART much earlier. Point of care HIV diagnosis will facilitate early diagnosis but must still be established in non-research settings. Also, despite progress in reducing vertical transmission, 160,000 newly infected infants were born in 2016. Vertical transmission during breast feeding remains an ongoing risk, with a high likelihood of late diagnosis [39]. We had previously noted that the majority of HIV+ infants already have advanced HIV disease by 12 weeks of age in a public program [40]. IRIS can also occur in children with asymptomatic HIV disease and high CD4 counts, as noted in our study. Although most ART regimens in our study are still widely used, the integrase strand inhibitors are being introduced and will become an important component of ART. Recent cohort studies suggest a higher rate of IRIS due to more rapid viral load reduction, [41] but this was not born out in a randomized study [42].

## Conclusions

IRIS occurs commonly in HIV-infected infants and young children. It is commonly, but not exclusively, seen in those with advanced HIV disease. Adequate screening for TB and CMV infection is essential to minimise unmasking IRIS. Although often benign and self-limiting, the consequences of IRIS can be severe. A better understanding of pathogenesis and identification of biomarkers to predict IRIS risk and assist diagnosis are needed.

## Supporting information

S1 Fig Local and regional BCG IRIS in an infant. (TIF)

S2 Fig A. Lateral chest radiograph showing perihilar infiltration in SID 1228. B. Contrast-enhanced Brain CT showing lesion surrounded by oedema in left parietal lobe of same participant.
(TIF)

S3 Fig T2-weighted Brain MRI from SID 1652 showing multiple granulomas surrounded by inflammatory oedema (white signal). (TIF)

S4 Fig A. Non-IRIS infectious and inflammatory events over time. B. Categories of non-IRIS infectious and inflammatory events.
(PDF)

S1 Table ART regimens. (DOCX)

S2 Table BCG IRIS. (DOCX)

S3 Table TB IRIS using INSHI criteria. A. Unmasking B.Paradoxical.
(DOCX)

S4 Table Deaths and relationship to IRIS. (DOCX)

S5 Table IRIS with severe morbidity or mortality. (DOCX)
