# Supplementary figures and images for: A prospective study of the immune reconstitution inflammatory syndrome (IRIS) in HIV-infected children from high prevalence countries
Source: PLoS One. 2019 Jul 1;14(7):e0211155. doi: 10.1371/journal.pone.0211155 (PMC6602181; doi:10.1371/journal.pone.0211155)

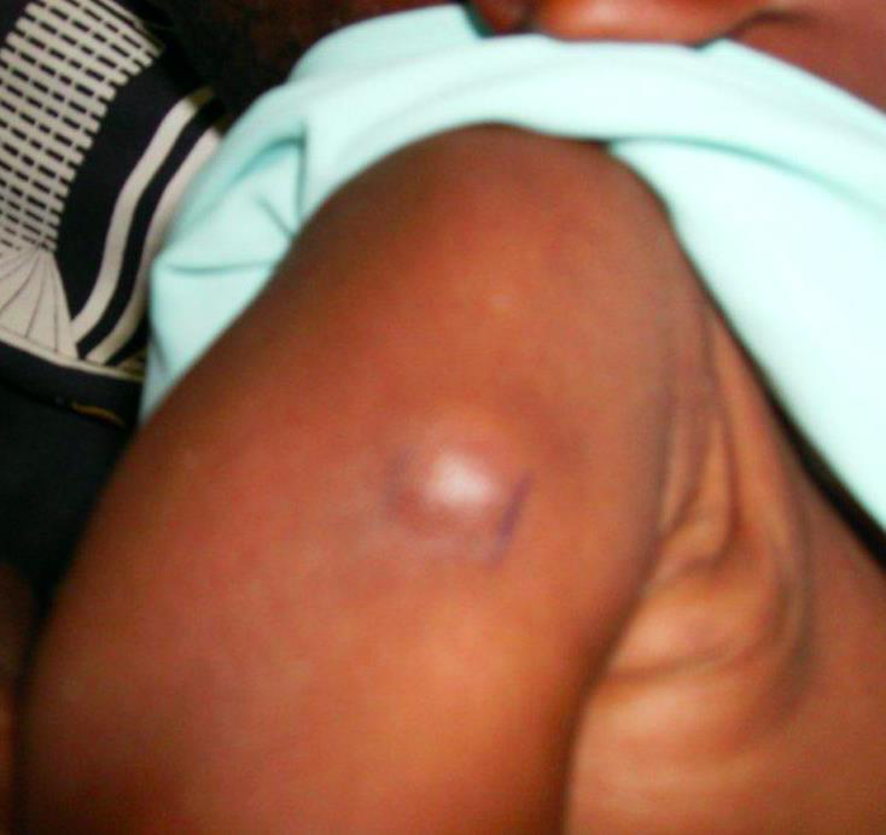

Supplement: S1 Fig — (TIF) [file pone.0211155.s001.tif]

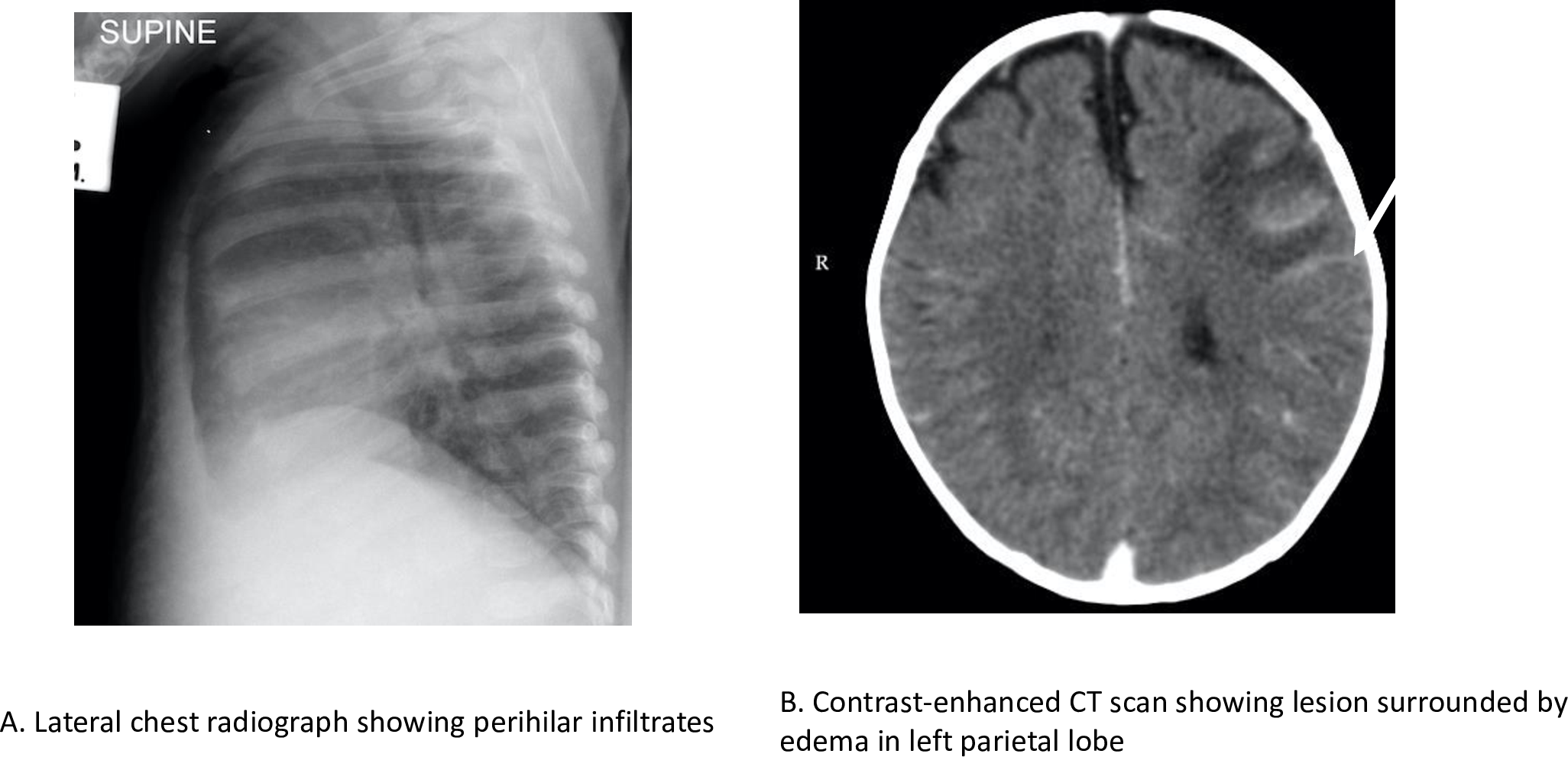

Supplement: S2 Fig — A. Lateral chest radiograph showing perihilar infiltration in SID 1228. B. Contrast-enhanced Brain CT showing lesion surrounded by oedema in left parietal lobe of same participant. (TIF) [file pone.0211155.s002.tif]

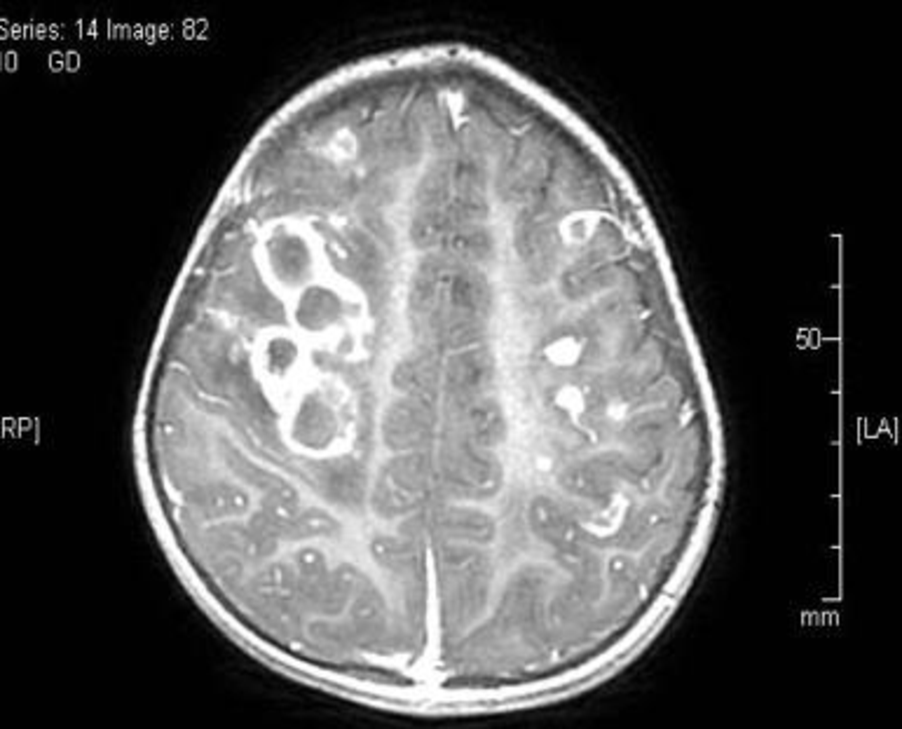

Supplement: S3 Fig — (TIF) [file pone.0211155.s003.tif]
