# Supplementary material for: A prospective study of the immune reconstitution inflammatory syndrome (IRIS) in HIV-infected children from high prevalence countries
Source: PLoS One. 2019 Jul 1;14(7):e0211155. doi: 10.1371/journal.pone.0211155 (PMC6602181; doi:10.1371/journal.pone.0211155)
Supplement: S4 Fig — A. Non-IRIS infectious and inflammatory events over time. B. Categories of non-IRIS infectious and inflammatory events. (PDF) [file pone.0211155.s004.pdf]

Figure 4A

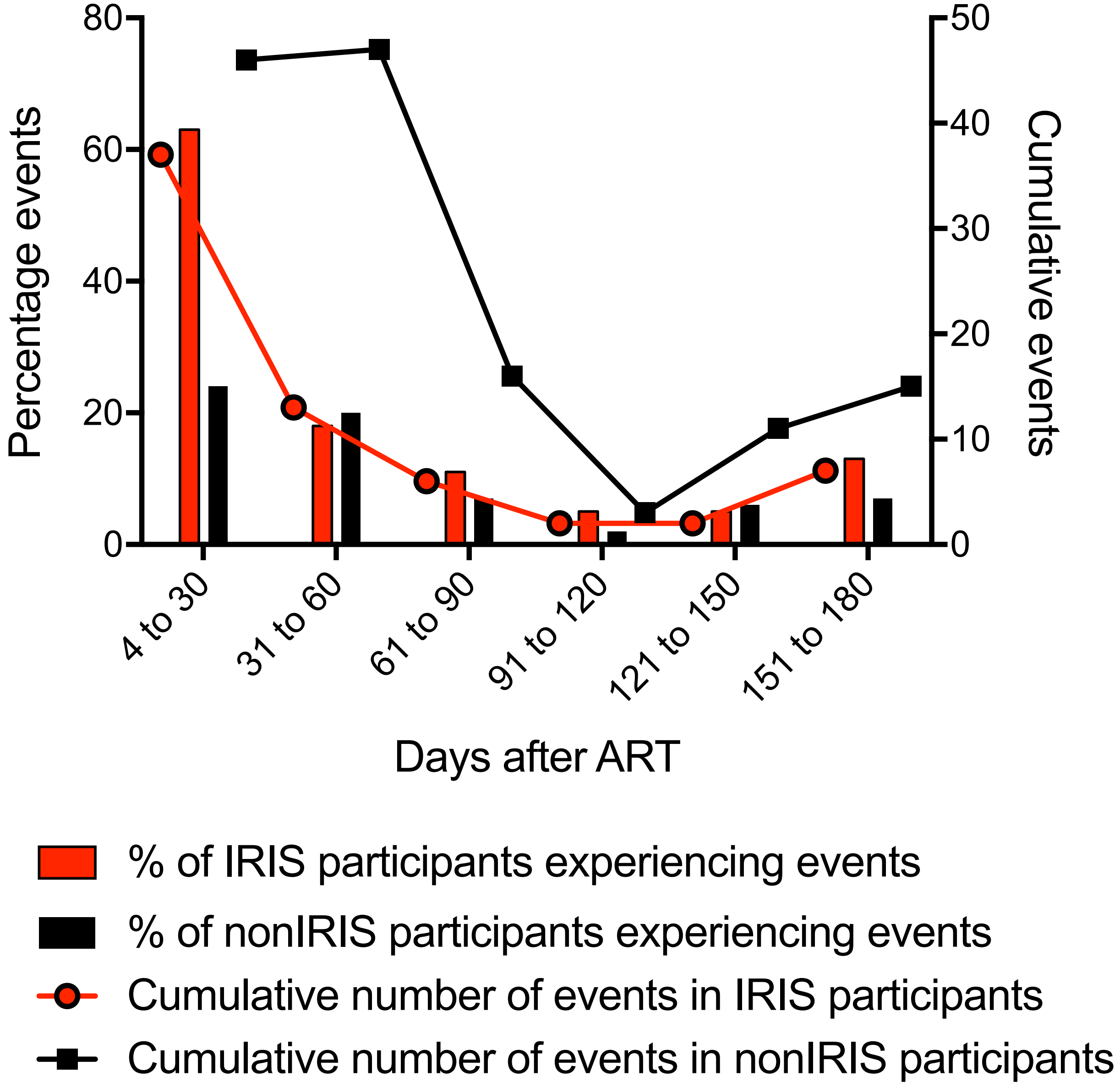
80
60
40
20
0
Percentage events
50
40
30
20
10
0
Cumulative events
4 to 30
31 to 60
61 to 90
91 to 120
121 to 150
151 to 180
Days after ART
% of IRIS participants experiencing events
% of nonIRIS participants experiencing events
Cumulative number of events in IRIS participants
Cumulative number of events in nonIRIS participants

Figure 4B

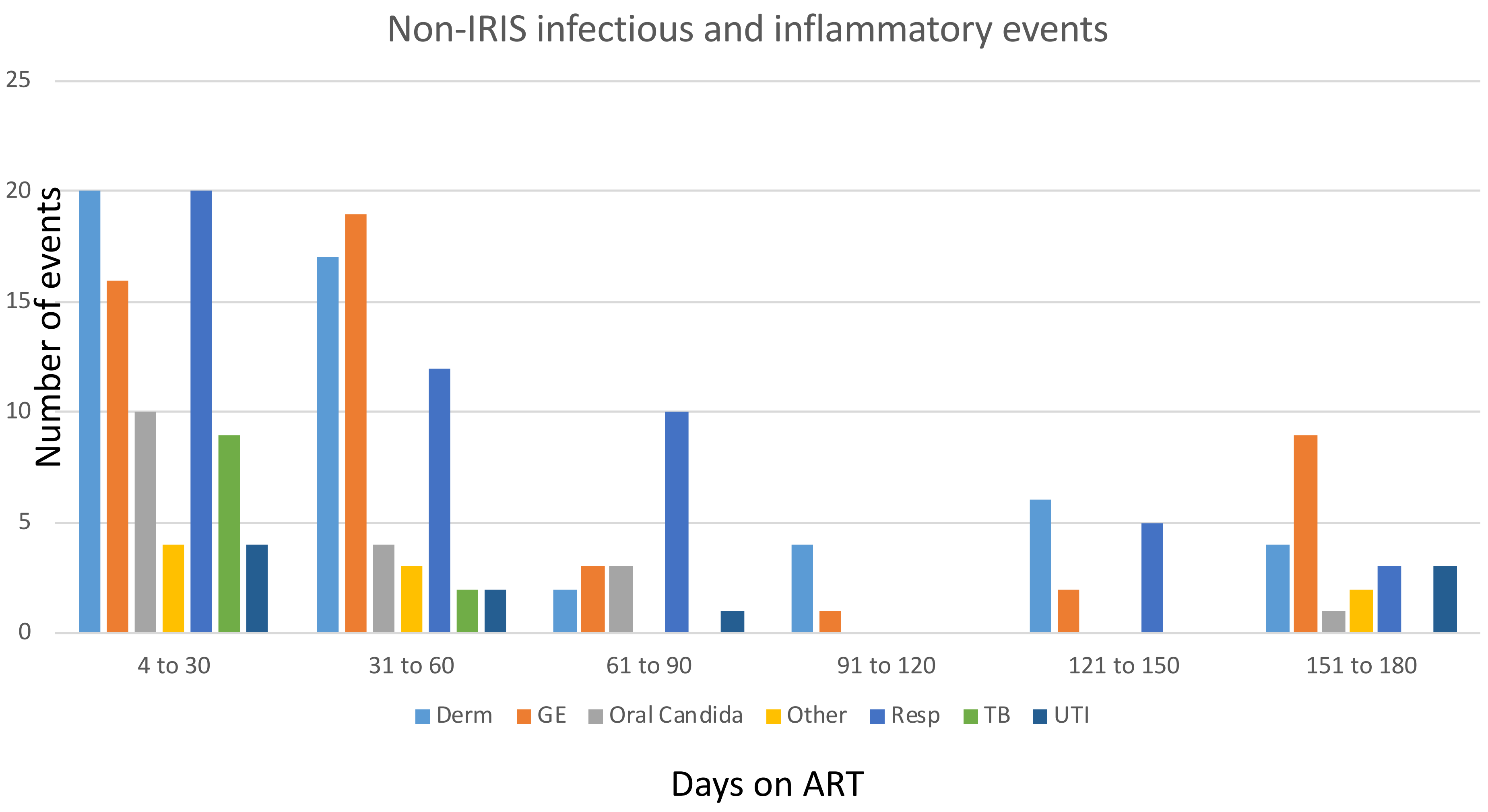
Non-IRIS infectious and inflammatory events
Number of events
25
20
15
10
5
0
4 to 30
31 to 60
61 to 90
91 to 120
121 to 150
151 to 180
Derm
GE
Oral Candida
Other
Resp
TB
UTI
Days on ART
