# Supplementary material for: A prospective study of the immune reconstitution inflammatory syndrome (IRIS) in HIV-infected children from high prevalence countries
Source: PLoS One. 2019 Jul 1;14(7):e0211155. doi: 10.1371/journal.pone.0211155 (PMC6602181; doi:10.1371/journal.pone.0211155)
Supplement: S1 Table — (DOCX) [file pone.0211155.s005.docx]

**S1 table. ART regimens**

| ART regimen | All (%)  198* | No IRIS (%)  160 | IRIS (%)  38 | P-value |
| --- | --- | --- | --- | --- |
| PI:  Lopinavir/ritonavir  + NRTIs   - ABC + LMV - ZDV + LMV - STV + LMV | 130 (65)   - 114 (57.6) - 10 - 6 | 103 (52.2)   - 90 - 10 - 3 | 27 (71.1)   - 24 - - - 3 | 0.57* |
| NNRTI:  Efavirenz + 2 NRTIs   - ABC + LMV - ZDV + LMV - STV + LMV   Nevirapine + NRTIs   - ABC + LMV - ZDV + LMV - STV + LMV - ZDV + LMV + ABC | 68 (34.3)    16   - 10 - 5 - 1   50 (25.3)   - - - - 38 - - 11 - - 1 | 57 (35.6)  11   - 6 - 4 - 1   44   - - - 34 - 9 - 1 | 11 (28.9)  5   - 4 - 1 - -   6   - - - 4 - 2 - - |  |
| 3 NRTIs   - ABC + LMV + ZDV | 2 | 2   - 2 | -   - - |  |

PI - Protease inhibitor; NNRTI - Non-nucleoside reverse transcriptase inhibitor; NRTI – nucleoside reverse transcriptase inhibitor; ABC – Abacavir; LMV – Lamivudine; ZDV – Zidovudine; STV - stavudine

Lopinavir/ritonavir-based versus NNRTI-based + 3 NRTIs*
