# Supplementary material for: A prospective study of the immune reconstitution inflammatory syndrome (IRIS) in HIV-infected children from high prevalence countries
Source: PLoS One. 2019 Jul 1;14(7):e0211155. doi: 10.1371/journal.pone.0211155 (PMC6602181; doi:10.1371/journal.pone.0211155)
Supplement: S2 Table — (DOCX) [file pone.0211155.s006.docx]

**S2 table. BCG IRIS**

| N | 21 |
| --- | --- |
| Age (months)* | 0.37 (0.26; 0.61) |
| On medication active against BCG prior to IRIS | 5# |
| Type of IRIS:  Local only  Regional only  Local and regional | 9 (43%)  6 (29%)  6 (29%) |
| Time to onset (weeks)* | 4 (1.9; 8) |
| Duration (weeks)* | 22 (10.9; 31) |
| Aspiration for symptomatic relief | 2 (9.5%) |

# Amikacin in one, amikacin plus isoniazid in one, rifampicin in one and INH plus rifampicin in two participants

*Median / IQR

One of 3 participants commencing IPT developed BCG IRIS

All sites used BCG from Statens Serum Institute, Denmark
