# Supplementary material for: A prospective study of the immune reconstitution inflammatory syndrome (IRIS) in HIV-infected children from high prevalence countries
Source: PLoS One. 2019 Jul 1;14(7):e0211155. doi: 10.1371/journal.pone.0211155 (PMC6602181; doi:10.1371/journal.pone.0211155)
Supplement: S3 Table — A. Unmasking B.Paradoxical. (DOCX) [file pone.0211155.s007.docx]

**S3 table. TB IRIS using INSHI criteria**

1. Unmasking TB IRIS

| **Case** | **Location of IRIS event** | **Onset IRIS**  **(ART days)** | **Plasma HIV RNA reduction**  **(copies/mm^3^)** | **Features of IRIS event** | **TB diagnosis** | **Comments** |
| --- | --- | --- | --- | --- | --- | --- |
| 1228 | CNS tuberculoma | 14 | Log 3.19 | Seizures.  CXR unchanged.  CT scan: intracerebral granulomas | PTB: History of coughing.  CXR - Hilar adenopathy and interstitial changes. | Anti-TB treatment commenced 40 days prior to ART |
| 1245 | Lung | 14 | Log 1.5 | Increased respiratory distress.  CXR – increased nodularity compared to baseline | Contact with TB source case (father) missed at baseline | At baseline, mild cough with CXR considered viral pneumonitis |
| 1246 | Abdomen | 62 | Log 0.42 | Progressive obstructive jaundice | Mantoux skin test 26 X16mm induration  PTB (pleural effusion) in mother when IRIS suspected.  Negative gastric aspirate culture | Off ART for 4 days preceding viral load at IRIS diagnosis. |
| 1256 | Lung | 38 |  | New cough and fever | Mantoux skin test converted from negative at baseline to 8mm induration  N | CXR: Consolidation and collapse right mid lobe and ligula  At baseline, was not suggestive of TB |
| 1652 | *CNS:  Meningitis &  granulomas | 53 | Log 3.5 | Raised ICP, seizures, depressed level of consciousness | N | Only supporting evidence was improvement on ant-TB therapy. |
| 1224 | Lung | 19 | Log 1.81 | Respiratory symptoms:  CXR: new perihilar and paratracheal lymphadenopathy | N | Baseline CXR normal |
| 1231 | Lung: | 62 | Log 1.69 | New respiratory symptoms  Positive Mantoux skin test – 15mm induration  CXR: Hilar nodes, patchy alveolar opacification right and left lower lobes | N | Baseline CXR normal |

B. Paradoxical TB IRIS

| **Case** | **Initial anatomical site for TB** | **Day on ART** | **Viral reduction** | **Paradoxical criteria** | | **Bacteriological**  **confirmation** |
| --- | --- | --- | --- | --- | --- | --- |
|  |  |  |  | **Major** | **Minor** |  |
| 1244 | Lung: hilar and alveolar | 14 | Log 2.44 | Left lower bronchus compression | Worsening respiratory symptoms | No |
| 1269 | Lung:  Pericardial  Cervical adenitis | 18 | Log 2.83 | Increased pericardial effusion. Increased left upper lobe consolidation (new CXR changes)  Cervical LN drained spontaneously | Worsening respiratory  symptoms | *M. tb* complex  Sensitive to RIF and INH |
| 1302* | Lung:  hilar LNs and parenchymal changes  Cervical adenitis | 14 | Log 2.51 | Increased size of parenchymal and hilar LNs  Increased size of cervical LNs and new right axillary LNs | Fever | *M. tb* complex |

INSHI - international network for study of HIV-associated IRIS; CNS – central nervous system; CXR – chest radiograph; PTB - pulmonary TB; *M. tb* – *M. tuberculosis*; LN – lymph nodes; RIF – rifampicin; INH – isoniazid; ICP – intracranial pressure

*Not from SU
