# Supplementary material for: A prospective study of the immune reconstitution inflammatory syndrome (IRIS) in HIV-infected children from high prevalence countries
Source: PLoS One. 2019 Jul 1;14(7):e0211155. doi: 10.1371/journal.pone.0211155 (PMC6602181; doi:10.1371/journal.pone.0211155)
Supplement: S4 Table — (DOCX) [file pone.0211155.s008.docx]

**S4 table. Deaths and relationship to IRIS**

| **Patient**  **(Age)** | **Baseline status** | **IRIS event** | **Cause and time of death** |
| --- | --- | --- | --- |
| IRIS related | | | |
| 1302  (5.7 years) | PTB and LN (right axillary and supra-clavicular) TB  Drug-sensitive *M.tb* on fine needle aspirate and sputum culture; on anti-TB therapy and prednisolone for 20 days  WAZ -0.31  VL log 5.4 copies/mm^3^  CD4 - 10 cells/mm^3^ | Paradoxical TB in lung parenchyma and LN on day 14 of ART | Developed extensive vasculitic skin disease in hospital and died on day 33 |
| IRIS present but unrelated | | | |
| 1518  (2.7 years) | Multi-lobe pneumonia (alveolar pattern on CXR).  WAZ -3.6  VL log 6.5 copies/mm^3^  CD4 count – 3 cells/mm^3^ | Oral candidiasis (inner lips, buccal mucosa and tongue, extending to pharynx on day 10 of ART | Invasive pneumococcal disease on day 35 |
| 1587  (3 months) | Preterm birth; gestation age - 35 weeks  Presented with failure to thrive.  WAZ -5.6  VL log 7.6 copies/mm^3^  CD4 count – 540 cells/mm^3^ (17.1%) | BCG – local and regional on day 10 of ART | Acute gastroenteritis after 3 months |
| No IRIS identified | | | |
| 1238  (4 months) | Oral candidiasis  WAZ -1.92  CD4 1489 cells cells/mm^3^ (12.7%)  Viral load log 6.5 copies/mm^3^ |  | Acute gastroenteritis after 6 weeks, hospitalized in rural hospital for 2 weeks and demised shortly after discharge |
| 1317  (1.3 years) | HIV encephalopathy, oral candidiasis, PTB, urinary tract infection  WAZ -1.34  CD4 161 cells/mm^3^ (6.7%)  VL log 4.2 copies/mm^3^ |  | Aspiration pneumonia and sepsis precipitated by pseudobulbar palsy after 12 weeks |
| 1299  (2.8 years) | WHO Stage 1  WAZ -0.25  CD4 1466 cells/mm^3^ (22.1%)  VL log 4.6 copies/mm^3^ |  | Died at home after 48 weeks; family did give information |
| 1376  (1.7 years) | Oral candidiasis, generalized lymphadenopathy, severe wasting  severe anemia (Hb 7.8 g/dL)  WAZ – 8.1  CD4 1883 cells/mm^3^ (32%)  VL log 5.6 copies/mm^3^ |  | Died at home on day 13, no further information |
| 1392  (4.1 years) | Cough and nasal discharge  WAZ -8.9  CD4 146 cells cells/mm^3^ (7%)  Viral load log 6 copies/mm^3^ |  | Died after 2.4 weeks; no additional information |
| 1397  (2 years) | History of severe recurrent pneumonia  WAZ -3.4  CD4 1321cells cells/mm^3^ (25%)  Viral load log 6 copies/mm^3^ |  | Died after 29 weeks at home of possible pneumonia |

PTB – pulmonary tuberculosis; CXR – chest radiograph; WAZ – weight for age Z-score

CXR – chest radiography
