# Supplementary material for: A prospective study of the immune reconstitution inflammatory syndrome (IRIS) in HIV-infected children from high prevalence countries
Source: PLoS One. 2019 Jul 1;14(7):e0211155. doi: 10.1371/journal.pone.0211155 (PMC6602181; doi:10.1371/journal.pone.0211155)
Supplement: S5 Table — (DOCX) [file pone.0211155.s009.docx]

**S5 table. IRIS with severe morbidity or mortality**

| **Patient**  **(Age)**  **(Site)** | **Baseline clinical and laboratory status** | **Preceding ART and other treatment** | **IRIS event** | **Presenting signs and symptoms for IRIS** | **HIV VL and CD4 response at time of IRIS** | **Investigations & management of IRIS** | **Course** |
| --- | --- | --- | --- | --- | --- | --- | --- |
| 1302  (5.7 years)  UKZN | PTB and LN (right axillary and supra-clavicular) TB  Drug-sensitive *M.tb* on fine needle aspirate and sputum culture;  WAZ -0.31  VL log 5.4 copies/mm^3^  CD4 - 10 cells/mm^3^ | ART day 14  Anti-TB therapy and prednisolone day 34 | Paradoxical TB in lung parenchyma and cervical LN | Fever, tender enlarged supraclavicular lymph node. | CD4 increased to 72 cells/mm^3^  Viral load declined to log 1.9 copies/mm^3^ | Increased opacities and widened mediastinum on CXR  Treated for sepsis  Prednisone added | Developed extensive vasculitic skin disease  Died on day 33 |
| 1228  0.61 years  SU | PTB: cough and perihilar infiltrates on CXR.  WAZ -3.63  CD4 count - 2077 cells/ml  Viral load log 6.8 copies/ml | ART day 13  Anti-TB treatment: day 53 | Unmasking CNS TB granulomas  (CNS lesions not suspected at baseline) | Complex focal seizure for an hour  Ring-enhancing lesions in right caudate and left parietal lobe (CT scan) | CD4 count increased to 2192 cells/ml  VL reduced to 3.61 log copies/ml | Anti-TB treatment: Eth added; dosage RIF, INH and PZA increased.  Prednisone added at 2mg/kg/day | Hospitalized for 8 months.  Residual developmental delay. |
| 1303  5.4 years  UKZN | Cryptococcal meningitis: blood culture positive X2; CSF positive for CRAG (bloody tap)  WAZ -0.86  CD4 count - 14 cells/ml  Viral load log 5.6 copies/ml | ART Day 95  CM: AmB for 2 weeks, fluconazole (12 mg/kg/day X weeks, then 6mg/kg daily  Anti-TB treatment: Rif/INH/PZA/EMB | Paradoxical Cryptococcal meningitis | Inability to walk for 2 days and weight loss  Culture and CRAG negative. | CD4 count increased to 106 cells/ml  VL reduced to <log 2.1 copies/ mm^3^ | Repeat CSF: culture and CRAG negative; 26 lymphocytes per mm^3^.  Fluconazole increased to 12mg/kg/day for 4 weeks  Prednisolone for 2 weeks | Hospitalized for 11 days  Fluconazole at 12mg/kg/day repeated with good response |
| 1255  1.4 years  SU | Bicytopenia (Low hematocrit and platelet count).  PTB (culture confirmed on day 16)  WAZ -2.34  CD4 count - 753 cells/ml  VL log 6.55 copies/ml  Anti-TB treatment preceded ART by 5 days; CXR unchanged from baseline, no abdominal lymph nodes on abdominal sonar) | ART Day 9  Anti-TB treatment: Day 14 | Unmasking CMV  Colitis | Severe bloody diarrhea, shocked, abdominal distension  CMV pp65 positive (count – 5 cells). CMV viral load -2722 copies/ml (log 3.43)  Abdominal sonar - thickened gall bladder and moderate ascites. | CD4 count decreased to 234 cells/ml  HIV VL reduced to log 3.8 copies/ml | Ganciclovir  Prednisolone | Required intensive care, inotropic support |
| 1282  5 months  SU | HIV encephalopathy.  WAZ -1.94  CD4 count - 876 cells/ml  VL RNA log 7 copies/ml  CMV pneumonia – Ganciclovir X 2 weeks until 4 days on ART (CMV viral load log 4.14 after completion) | ART: Day 19  Anti-TB treatment: Day 26  (TB never confirmed) | Paradoxical CMV colitis and pneumonitis  (also BCG IRIS) | Bloody diarrhea and shock  Respiratory failure  CMV viremia confirmed (log 3.74 copies/ml)  CXR suggestive of CMV | CD4 count decreased to 215 cells/ml  VL reduced to log 4 copies/mm^3^ | Ganciclovir | Required intensive care, inotropic support |
| 1246  8 months  SU | Suppurative otitis media for 3 months, then right middle lobe pneumonia and meningitis.  WAZ 0.2  CD4 count – 931 cells/mm^3^  VL log 5.95 copies/mm^3^ | ART: Day 40  ART: Day 8 | Unmasking  abdominal TB + biliary obstruction  ALT 48, AST 79, ALP 1434 GGT 1229, Total bilirubin 144, conjugated 109  (Also HPPE IRIS) | Obstructive jaundice and enlarged liver: biopsy showed bridging necrosis suggesting biliary obstruction.  TB: strongly positive TST, Mother had pleural effusion & responded to anti-TB treatment: | CD4 count decreased to 435 cells/mm^3^  VL reduced to log 5.59 copies/ mm^3^ | Anti-TB treatment Prednisone | ART interruptions due to severity of biliary obstruction  Hospitalized for 6 months  Bile lake seen on MRI, gall bladder destroyed. Porto-jejunostomy successful |
| 1652  1.8 years  BJMC | WAZ 0.2  CD4 count – 3739 cells/mm^3^  Plasma HIV RNA log 5.65 copies/mm^3^ | Day 53 | Possible unmasking TB granulomas  (On basis of response to anti-TB meningitis treatment) | Fever, vomiting, rapid worsening level of consciousness and raised ICP  CSF 700 cells (Neutrophils 85%), protein 20mg/dl, glucose 40mg/dl  MRI – multiple ring-enhancing lesions and lepto-meningeal enhancement suggesting TB granulomas | CD4 count decreased to 2403 cells/mm^3^  VL reduced to log 2.15 copies/ mm^3^ | CSF culture –ve for TB, fungi, CRAG  CSF PCR negative for HSV, serology negative for HSV 1 and 2.  Antibody negative for toxoplasmosis in serum and CSF, Serum negative for cysticercosis. Rapid malaria antigen test negative  All TB studies negative including repeat Mantoux at Week 24 (day 136) | ICU admission.  Seizures  Residual hemiparesis  Anti-TBM* treatment initiated after 7 days in ICU followed by improvement |

PTB – pulmonary tuberculosis; WAZ – weight for age Z-score; Ethionamide Eth; Isoniazid (INH), rifampicin (RIF), pyrazinamide (PZA); LN- lymph nodes; CXR – chest radiograph; AmB – Amphotericin B; CRAG – cryptococcal antigen; GER – gastro-esophageal reflux; WAZ – weight for age Z-score; TST – Tuberculin skin test; CXR – chest radiograph; ICU – intensive care unit; ICP – intracranial pressure;

CNS – central nervous system; TBM – tuberculous meningitis; GCV – ganciclovir; MRI – magnetic resonance imaging
